# The effect of integrating situational frustration education into physical education teaching on the psychological resilience of junior high school students: a 16 week parallel controlled teaching experimental study

**DOI:** 10.3389/fpsyg.2026.1848049

**Published:** 2026-07-03

**Authors:** Qiaoyan Yu, Huiping Zhang, Hongcui Yuan, Chenliang Deng

**Affiliations:** 1 School of Physical Education, Chengdu Sport University, Chengdu, China; 2 No. 2 Middle School of Xuyong County, Luzhou, China; 3 Sports Department, University of Electronic Science and Technology of China, Chengdu, China

**Keywords:** junior high school students, physical education, psychological resilience, self-determination theory, situational frustration education, teaching experiment

## Abstract

**Objective:**

Insufficient psychological resilience among adolescents has become a critical public health concern that urgently needs to be addressed in China’s basic education sector. Physical education serves as a natural vehicle for implementing frustration education and enhancing adolescents’ psychological resilience. Grounded in Self-Determination Theory (SDT) and indigenous frustration education theory, this study aims to construct a systematic curriculum system integrating situational frustration education into junior high school physical education, and examine its intervention effect on students’ psychological resilience through a 16-week parallel controlled teaching experiment, so as to provide theoretical basis and practical reference for junior high school physical education to achieve the goal of integrated development of physical and mental education.

**Methods:**

Using a cluster sampling method, a total of 114 students from two parallel Grade 8 classes at No. 2 Middle School of Xuyong County, Luzhou City, Sichuan Province were recruited as participants. One class was randomly assigned to the experimental group (*n* = 57) and the other to the control group (*n* = 57). The experimental group received physical education integrated with situational frustration education, while the control group received conventional physical education. The Resilience Scale for Chinese Adolescents (RSCA) was administered to both groups before and after the experiment. Independent-samples t-tests, paired-samples t-tests, repeated-measures mixed ANOVA, and analysis of covariance (ANCOVA) were performed using SPSS 29.0 software.

**Results:**

(1) Repeated-measures mixed ANOVA revealed a significant Time × Group interaction effect on the total psychological resilience score (*F* = 6.896, *p* = 0.010, partial η^2^ = 0.058). ANCOVA results showed that after controlling for baseline scores, the post-test total psychological resilience score of the experimental group was significantly higher than that of the control group (*p* < 0.001). (2) In the experimental group, students’ scores on the dimensions of Emotion Control, Goal Focus, Interpersonal Assistance, and Positive Cognition improved significantly after the experiment (*p* < 0.05), while no significant change was observed in the Family Support dimension (*p* > 0.05). (3) In the control group, students’ scores on Emotion Control, Goal Focus, and Positive Cognition improved significantly (*p* < 0.05), while no significant changes were found in Interpersonal Assistance and Family Support (*p* > 0.05). (4) Situational frustration education significantly improved psychological resilience in both male and female junior high school students, with no significant gender difference in the intervention effect (*p* > 0.05).

**Conclusion:**

Integrating situational frustration education into physical education can significantly improve the overall psychological resilience of junior high school students, and its effect is significantly better than that of conventional physical education. It exerts differential improvement effects on various dimensions of psychological resilience, significantly enhancing students’ abilities in emotion control, goal focus, interpersonal assistance, and positive cognition, making it an effective practical pathway for implementing mental health education objectives in junior high school physical education.

## 1 Introduction

With the in-depth implementation of the Healthy China 2030 Planning Outline, adolescent mental health has become one of the core components of Healthy China construction. The World Health Organization (WHO) explicitly defines health as “a state of complete physical, mental and social well-being and not merely the absence of disease or infirmity.” This definition has established the core goal of integrated development of physical and mental education for school education in the new era. Currently, junior high school students in China are in the early adolescence stage of dramatic physical and psychological changes, while simultaneously facing multiple growth pressures such as academic competition, interpersonal adaptation, and self-identity. As a result, mental health problems are showing a trend of high incidence and younger age of onset. A study by Xu and Li (2023) indicated that approximately 1 in 6 middle school students in China have or have had suicidal ideation, and suicide has become the leading cause of death among adolescents in China. Insufficient psychological resilience and low frustration tolerance are the core triggers of adolescent mental health crises.

Psychological resilience refers to an individual’s ability to psychologically recover and adapt positively when facing adversity, stress, and frustration, and it serves as a core protective factor for adolescent mental health (Hu and Gan, 2008). Extensive research has confirmed that psychological resilience not only effectively buffers the negative impacts of stressful events on adolescent mental health but also positively predicts their academic achievement, social adaptation ability, and long-term life development (Qiu et al., 2025). Therefore, how to enhance adolescents’ psychological resilience through systematic educational interventions has become a research hotspot and practical priority in the field of basic education. In recent years, the international sport psychology field has increasingly focused on the relationship between frustration of basic psychological needs and psychological resilience. Self-Determination Theory (SDT) posits that autonomy, competence, and relatedness are three fundamental psychological needs of human beings. When these needs are moderately satisfied, individuals exhibit higher motivation, well-being, and psychological resilience; conversely, excessive frustration of these needs leads to negative emotions such as anxiety and depression (Trigueros and García-Mas, 2025). A further study by Fernandez-Ortega et al. (2024) confirmed that moderate and controllable need frustration can promote individual adaptive development, while unguided and excessive frustration has destructive effects. This theory provides a contemporary international perspective for understanding the mechanism of frustration education. Frustration education, an educational concept with Chinese characteristics, is the core pathway to enhancing adolescent psychological resilience. Wu (1994) was the first to systematically elaborate the theoretical system of frustration education in China, pointing out that frustration education is an educational activity that cultivates individuals’ ability to correctly recognize, prevent, and manage frustration by presenting frustration factors in human social activities. Its core is to guide individuals to dialectically view the duality of frustration, leverage its positive educational value, and avoid its negative harms. Yu (2007) further clarified that the essence of frustration education is not to artificially create frustration, but to guide learners to scientifically understand failure, make reasonable attributions, minimize the negative effects of frustration, and draw growth nutrients from it to ultimately achieve self-development.

However, frustration education in primary and secondary schools currently faces practical dilemmas of nihilization, deformalization, and formalism. Issues such as cognitive deviations between home and school, lack of educational principles, and single educational methods have made it difficult for frustration education to be effectively implemented (Zhang, 2019). Physical education is a natural vehicle for frustration education, characterized by its competitiveness, practicality, and challenging nature. Students inevitably encounter authentic frustration experiences during skill learning and competitive matches (Yang, 2005; Wang et al., 2001). Nevertheless, most frustrations in existing physical education occur randomly, lacking systematic design and guidance, making it difficult to maximize their educational value. A comprehensive review of existing research reveals that domestic and international scholars have conducted extensive discussions on frustration education in physical education, but three core limitations remain: First, existing studies are mostly theoretical speculations, and empirical studies are predominantly cross-sectional surveys, with a scarcity of longitudinal intervention experiments targeting junior high school students. Second, the implementation of frustration education is mostly fragmented application of methods, lacking systematic curriculum design deeply integrated with physical education content, and failing to effectively connect with contemporary international psychological theories such as SDT. Third, few studies have explored the differential effects of situational frustration education on various dimensions of junior high school students’ psychological resilience, resulting in insufficient refined analysis of intervention effects.

Against this background, this study constructs a systematic curriculum system integrating situational frustration education into junior high school physical education based on embodied cognition theory, frustration duality theory, psychological resilience process theory, and SDT. Through a 16-week parallel controlled teaching experiment, it systematically examines the intervention effects on the overall level and various dimensions of junior high school students’ psychological resilience. The core objective of this study is to verify the enhancement effect of situational frustration education integrated into physical education on junior high school students’ psychological resilience and explore its differential effects on various dimensions of psychological resilience. Four research questions are proposed based on the study objective: Can situational frustration education integrated into physical education significantly improve the overall psychological resilience of junior high school students? Does situational frustration education have differential enhancement effects on the five dimensions of junior high school students’ psychological resilience? Does physical education integrated with situational frustration education have a superior intervention effect compared to conventional junior high school physical education? Are there significant gender differences in the intervention effect of situational frustration education?

The theoretical significance of this study lies in combining indigenous frustration education theory with international SDT to construct a more explanatory theoretical framework and clarify the underlying mechanisms through which physical education promotes the development of adolescent psychological resilience. The practical significance lies in providing an operational practical pathway for junior high school physical education to implement mental health education objectives and offering new ideas and methods for the effective implementation of frustration education in primary and secondary schools.

## 2 Theoretical foundation and research hypotheses

### 2.1 Definition of core concepts

#### 2.1.1 Situational frustration education

In this study, situational frustration education is defined as an educational model grounded in frustration education theory and Self-Determination Theory (SDT). Based on Vygotsky’s Zone of Proximal Development (ZPD) (Wang, 2000), it creates hierarchical, authentic, and interactive moderate frustration scenarios in teaching, embeds frustration cognitive education, emotion regulation skills training, and frustration-resistant behavior cultivation into the entire teaching process, and guides students to correctly understand frustration and master coping strategies through embodied experiences, ultimately enhancing psychological resilience. Its core characteristics are: (1) deep integration of frustration scenarios with teaching content, rather than artificially creating frustration detached from teaching; (2) hierarchical progression of frustration difficulty in line with students’ physical and mental development laws; (3) realization of a complete educational closed loop of “frustration experience––cognitive reconstruction––skill acquisition––behavior reinforcement”; (4) control of frustration intensity within the scope of “moderate need frustration” to avoid harm to students caused by excessive frustration.

#### 2.1.2 Integration into physical education

This refers to the deep integration of the objectives, content, and methods of situational frustration education with the teaching content of the junior high school Physical Education and Health curriculum (including track and field, ball games, gymnastics, high school entrance examination physical education items, etc.), rather than additional training independent of physical education. On the basis of ensuring the realization of the core objectives of physical education (motor skill teaching and physical fitness improvement), it simultaneously achieves the objectives of frustration education and psychological resilience cultivation, realizing the organic unity of “skill acquisition” and “character cultivation,” as well as “physical education” and “mental education.”

#### 2.1.3 Psychological resilience

This study adopts the concept proposed by Hu and Gan (2008), defining psychological resilience as adolescents’ ability to adapt positively when facing adversity, stress, and frustration, which is a core indicator for measuring adolescents’ mental health. This concept includes five core dimensions: (1) Goal Focus: The ability to persist in goals, make plans, and concentrate on solving problems when facing adversity. (2) Emotion Control: The ability to regulate emotions, control impulses, and alleviate negative emotions when facing frustration. (3) Positive Cognition: The ability to dialectically view frustration and discover growth opportunities from adversity. (4) Family Support: The belief that one can obtain emotional support and material help from family when facing frustration. (5) Interpersonal Assistance: The ability to obtain help from peers and others and establish good interpersonal relationships when facing adversity.

### 2.2 Core theoretical foundations

#### 2.2.1 Duality theory of frustration and essence theory of frustration education

Wu (1994) systematically proposed the duality theory of frustration, pointing out that frustration has three core characteristics: objectivity and subjectivity, certainty and uncertainty, and positive and negative values. From the perspective of educational value, frustration has both harmful and positive values: on the one hand, frustration hinders the normal activities of the subject and triggers negative emotions; on the other hand, if the subject can correctly understand and manage frustration, it has educational values such as promoting cognitive development, testing survival ability, and stimulating individual potential. This theory lays the core logic for this study: frustration education is not about artificially creating frustration, but about guiding students to understand the duality of frustration through reasonable scenario design and maximizing its positive educational value. Yu (2007) further elaborated the essence theory of frustration education, arguing that the core of frustration education is not to make students accept failure, but to guide learners to dialectically understand failure, make scientific attributions for failure, minimize the negative effects of failure, and draw growth nutrients from it. This theory clarifies the educational objective of this study: the core of situational frustration education is not to let students experience failure, but to teach them methods to cope with failure.

#### 2.2.2 Self-determination theory and moderate need frustration

Self-Determination Theory (SDT) posits that autonomy, competence, and relatedness are three fundamental psychological needs of human beings, and the degree of need satisfaction directly affects individuals’ motivation, well-being, and psychological resilience (Trigueros and García-Mas, 2025). Recent studies have shown that needs are not binary states of “satisfaction” and “frustration”; moderate and controllable need frustration can promote individual adaptive development. When students encounter moderate skill challenges in physical education, their need for competence is temporarily frustrated, but after overcoming difficulties through effort, they will gain stronger competence and self-efficacy (Fernandez-Ortega et al., 2024). This theory provides important guidance for the frustration scenario design of this study: the difficulty of frustration scenarios must be controlled within students’ ZPD, neither too easy to lead to insufficient need satisfaction nor too difficult to lead to excessive need frustration. Only moderate and controllable frustration can become a positive factor promoting the development of psychological resilience.

#### 2.2.3 Process theory of psychological resilience

The process theory of psychological resilience proposed by Richardson (2002) holds that psychological resilience is not an innate static trait, but a dynamic development process of interaction between individuals and the environment. When facing adversity, individuals will adjust their cognitive and behavioral patterns by activating their internal protective factors (such as emotion regulation ability, positive cognition, social support, etc.), and ultimately achieve psychological recovery and growth. This theory provides the core idea for the intervention design of this study: by creating controllable hierarchical frustration scenarios, guide students to repeatedly activate and strengthen internal protective factors, and gradually improve psychological resilience.

#### 2.2.4 Embodied cognition theory

Merleau-Ponty’s embodied cognition theory points out that cognition is not a pure brain thinking activity, but a product of the interaction between the body and the environment, and bodily experience is the basis for the formation and development of cognition (Zhang et al., 2021). Traditional frustration education mostly relies on classroom lectures, making it difficult for students to form authentic cognition and profound experiences. In contrast, physical education takes physical activity as the core. Students experience frustration and cope with challenges in embodied sports practice, which allows them to internalize frustration-resistant knowledge and skills into their own stable psychological traits. This is the core advantage of integrating situational frustration education into physical education.

#### 2.2.5 Bandura’s social learning theory

Bandura's (1977) social learning theory holds that self-efficacy is the core driving force for the improvement of individual psychological resilience, and successful experiences, role modeling, and peer support are the key paths to enhancing self-efficacy. In the teaching program of this study, students gain successful experiences through hierarchical task design, form vicarious reinforcement through role modeling by teachers and excellent students, and obtain peer support through group cooperation, thereby effectively improving students’ self-efficacy.

#### 2.2.6 Vygotsky’s zone of proximal development theory

Vygotsky proposed that students’ development has two levels: the current development level and the potential development level that can be achieved through adult guidance and individual effort. The gap between the two is the “Zone of Proximal Development (ZPD).” Education should guide students to cross the ZPD through setting appropriate challenges to achieve continuous improvement of abilities. This theory provides the core principle for the frustration scenario design of this study: the difficulty of frustration scenarios must be within students’ ZPD, that is, “within reach with a little effort,” to ensure the appropriateness and effectiveness of frustration education (Sun, 2007).

### 2.3 Review of domestic and international research status

#### 2.3.1 Research on frustration education and adolescent psychological development

Foreign research on frustration and frustration tolerance started early. In the 1930s, Dollard et al. (1939) first proposed the “frustration-aggression” theory, revealing the negative effect of frustration triggering aggressive behavior. Subsequently, Amsel (1958) proposed the “frustration effect” through the classic double-runway experiment, confirming that frustration can stimulate individual behavioral motivation under certain conditions. Since the 1990s, with the rise of positive psychology, the research focus has shifted to the positive cultivation of frustration tolerance and psychological resilience. In recent years, research on frustration of basic psychological needs under the framework of SDT has become a hotspot. A meta-analysis by Trigueros and García-Mas (2025) showed that moderate need frustration is positively correlated with psychological resilience, while excessive need frustration is negatively correlated with psychological resilience. Domestic research on frustration education began in the 1990s. Wu (1994) was the first to systematically construct the theoretical system of frustration education in China. Subsequently, domestic scholars have conducted extensive research on the connotation, value, practical dilemmas, and implementation paths of frustration education (Yu, 2007; Zhang, 2019). Overall, domestic theoretical research on frustration education is relatively mature, but its integration with international frontier theories still needs to be strengthened.

#### 2.3.2 Applied research on frustration education in physical education

The integration of physical education and frustration education is a hotspot in domestic and international research. Foreign research mainly focuses on the mechanism of physical activity on adolescents’ psychological resilience. A qualitative study by White and Bennie (2015) found that training challenges and competition frustrations in gymnastics can effectively cultivate adolescents’ resilience behaviors. A meta-analysis by Qiu et al. (2025) confirmed that physical activity is significantly positively correlated with the psychological resilience of adolescent students. Domestic scholars Yang (2005) were the first to explore the application of frustration education in competitive physical education. A teaching experiment by Jia (2010) confirmed that integrating outward bound training into physical education can significantly improve college students’ frustration tolerance. However, existing research still has obvious limitations: first, most empirical studies focus on college students, with a scarcity of longitudinal intervention studies targeting junior high school students; second, the implementation of frustration education is mostly fragmented application of methods, lacking systematic curriculum design; third, few studies have paid attention to the differential effects of frustration education on various dimensions of psychological resilience.

#### 2.3.3 Integrated research on situational teaching in physical and mental health education

The core of situational teaching is to stimulate students’ emotional experience and realize the integration of knowledge, emotion, intention, and behavior by creating authentic and vivid teaching scenarios. Domestic and international studies have confirmed that situational teaching has significant application effects in physical education and mental health education (Gao, 2020). However, existing research that deeply integrates situational teaching with frustration education, constructs a systematic curriculum system, and verifies its effects through rigorous experiments is still relatively scarce.

### 2.4 Research hypotheses

Based on the above theoretical foundation and research status, this study proposes the following research hypotheses:

*H1:* Integrating situational frustration education into physical education can significantly improve the overall psychological resilience of junior high school students.

*H2:* Integrating situational frustration education into physical education has differential enhancement effects on the five dimensions of junior high school students' psychological resilience. Specifically, it has significant enhancement effects on the dimensions of Emotion Control, Goal Focus, Interpersonal Assistance, and Positive Cognition, but no significant enhancement effect on the Family Support dimension.

*H3:* Compared with conventional junior high school physical education, physical education integrated with situational frustration education has a more significant enhancement effect on junior high school students' psychological resilience.

*H4:* Integrating situational frustration education into physical education has significant enhancement effects on the psychological resilience of both male and female junior high school students, and there is no significant gender difference in the enhancement effect.

## 3 Participants and methods

### 3.1 Participants

This study employed a cluster sampling method to recruit students from two parallel Grade 8 classes at No. 2 Middle School of Xuyong County, Luzhou City, Sichuan Province, aged 13–14 years. This stage represents early adolescence, a critical transitional period for physical and psychological development and a golden period for shaping psychological resilience (Zhang, 2019). Based on the experimental design, sample size estimation was performed using G*Power 3.1 software. With a statistical power (1-*β*) of 0.8, a significance level *α* of 0.05, and an effect size d of 0.5, the required minimum sample size was calculated to be 102 participants.

A total of 114 students were finally included in the study. One class was randomly assigned to the experimental group (*n* = 57; 29 males, 28 females; mean age = 13.44 ± 0.50 years), and the other to the control group (*n* = 57; 33 males, 24 females; mean age = 13.47 ± 0.50 years). Before the experiment, independent-samples t-tests and chi-square tests were conducted to compare the demographic data, total psychological resilience scores, and pre-test scores of each dimension between the two groups. The results showed good baseline homogeneity between the two groups (all *p* > 0.05), indicating valid experimental grouping. Detailed participant characteristics are presented in Table 1.

**Table 1:** Comparison of baseline data between the experimental group and the control group (M ± SD).

Indicators	Experimental group (*n* = 57)	Control group (*n* = 57)	*t*/*χ*^2^	*p*
Sex (male/female)	29/28	33/24	0.747	0.456
Age (years)	13.44 ± 0.50	13.47 ± 0.50	−0.373	0.71
Height (cm)	161.32 ± 6.40	159.56 ± 6.79	1.421	0.158
Weight (kg)	50.09 ± 9.82	48.64 ± 7.60	0.881	0.38
BMI	19.16 ± 3.02	19.08 ± 2.49	0.156	0.877
Emotion control	18.25 ± 4.16	17.82 ± 4.26	0.534	0.594
Goal focus	16.61 ± 4.18	16.79 ± 4.73	−0.21	0.834
Interpersonal assistance	19.35 ± 4.67	18.68 ± 5.87	0.671	0.503
Positive cognition	14.60 ± 3.54	13.84 ± 4.09	1.053	0.294
Family Support	19.61 ± 4.55	18.18 ± 4.99	1.607	0.111
Total Psychological resilience score	88.42 ± 13.34	85.32 ± 16.01	1.125	0.263

*p* > 0.05 indicates no statistically significant difference; *p* < 0.05 indicates a statistically significant difference; *p* < 0.01 indicates a highly statistically significant difference.

Inclusion criteria: (1) Regular enrolled Grade 8 students at the school, not sports special students; (2) Physically healthy, no major physical diseases or exercise contraindications, able to participate normally in physical education activities; (3) No prior systematic frustration education or psychological resilience training before the experiment; (4) Students and their parents provided informed consent and voluntarily participated in the study by signing an informed consent form.

Exclusion criteria: (1) Students with chronic diseases, physical disabilities, asthma, heart disease, or other conditions preventing normal participation in physical activities; (2) Students who had received psychological counseling, psychotherapy, or taken psychotropic medications recently; (3) Students with a history of self-harm, suicidal ideation, or post-traumatic stress disorder (PTSD); (4) Students who transferred schools or were absent for more than 4 class hours during the experiment.

This study was approved by the Ethics Review Committee of Chengdu Sport University (Approval No. CDSU-IRB-2025-199). All research procedures complied with the ethical requirements of the Declaration of Helsinki.

### 3.2 Methods

#### 3.2.1 Teaching experiment method

A parallel controlled pretest-posttest experimental design was adopted. The experiment lasted for 16 weeks from September to December 2025, with 3 class hours per week (45 min per class), totaling 48 class hours. Among them, the first 3 class hours were used for the pre-experiment test, the last 3 class hours for the post-experiment test, and the remaining 42 class hours for actual teaching intervention.

(1) Experimental Variable Control

Independent variable: Whether situational frustration education was integrated into physical education.

Dependent variable: Changes in the overall level of psychological resilience and scores of its five dimensions among junior high school students.

Control variables: ① Teaching instructor: Both classes were taught by the same physical education teacher; ② Teaching duration: The number of class hours, weekly class frequency, and duration per class were identical for both groups; ③ Teaching content: The core physical education content was identical for both groups, with differences only in teaching methods and scenario design; ④ External interference: Before the experiment, communication was conducted with the head teachers and parents of both classes, requesting no additional mental health education activities during the experiment; ⑤ Standardized teaching: To reduce experimenter bias (Edlund et al., 2021), the teacher strictly followed pre-developed standardized lesson plans. Two professional teachers randomly observed 1 class per week to ensure consistency in the teaching processes of both groups.

(2) Experimental Intervention Programs

Control group intervention program: The conventional teaching program based on the People’s Education Press (PEP) Physical Education and Health textbook for Grade 8 was adopted. The teaching content included four modules: track and field, volleyball, gymnastics, and one-minute rope skipping. Traditional teaching methods such as explanation, demonstration, and decomposition practice were used, following the teaching process of “opening part––warm-up part––main part––closing part.” No additional frustration scenarios were set, and no systematic frustration cognition or frustration resistance skill training was conducted during teaching.

Experimental group intervention program: On the basis of maintaining identical teaching content and duration as the control group, the situational frustration education curriculum system constructed in this study was adopted, embedding hierarchical frustration scenarios into the entire teaching process. The curriculum design followed four principles: ① People-oriented and individualized teaching principle; ② Moderation principle; ③ Integration principle of skill learning and frustration resistance cultivation; ④ Principle of strictness with kindness.

The specific teaching content and situational frustration education design for the experimental group are presented in Table 2. Teaching methods included explanation and demonstration, cooperative learning, situational teaching, role modeling, and psychological skill training. The teaching process for each class was: “Opening part (2 min)––Warm-up part (8 min)––Main part (30 min: skill explanation and demonstration––situational frustration task practice––group discussion and reflection––teacher guidance and frustration resistance skill explanation––second practice and improvement)––Closing part (5 min: emotion regulation and class summary).”

(3) Expert Validity Test of the Experimental Program

**Table 2:** Teaching content and situational frustration education design of the experimental group.

Items	Physical education and health curriculum content	Application and purpose of situational frustration education	Core frustration elements	Dimensions of psychological resilience improvement
Track and field	Fixed-distance variable pace running	Application: During the fixed running distance, the teacher suddenly changes the original allocation of “sprint segments” and “jogging segments” with a whistle, breaking the inertial expectation of the body and mind. Purpose: To train students’ ability to quickly respond to changes and adjust rhythm under fatigue state.	Goal obstacles and uncertainty. Progressive difficulty and repetitive failure.	Emotion control, goal focus
	Fixed-time variable pace running	Application: Alternating fast and slow running within the specified total time. In the second half of the run (when physical strength declines), students are required to simultaneously memorize and repeat short numbers, and the specified time to complete the same distance is gradually shortened. Purpose: To simulate high-pressure scenarios and train the ability to maintain cognitive focus and task execution when physiological resources are consumed.	Goal obstacles and uncertainty. Progressive difficulty and repetitive failure.	Emotion control, goal focus
	Obstacle run	Application: Temporarily change the obstacle passing methods before the start or during the race, adopt a team relay competition format, where individual mistakes will add to the total time of the whole team. Purpose: To cultivate decision-making ability in response to emergencies, and stimulate the motivation of individual efforts through team responsibility.	Goal obstacles and uncertainty. Social evaluation and responsibility pressure.	Emotion control, goal focus, interpersonal assistance
Volleyball	Digging and passing	Application: Set stepped continuous success goals (two-person paired digging from 10 consecutive times, 20 times to 30 times), and counting needs to restart immediately after a mistake. Purpose: To accumulate “mastery experience” of controlling tasks through minor improvements in the cycle of “near-success––failure––re-attempt,” and cultivate persistence.	Progressive difficulty and repetitive failure. Social evaluation and responsibility pressure.	emotion control, positive cognition, interpersonal assistance
	Serving and spiking	Application: Create a “key point” scenario, announce that “the next point is worth double” in the team competition, designate students to perform serving or spiking, whose personal performance directly determines the team’s score. Purpose: To place personal skills under public pressure related to team success or failure, and exercise the psychological quality of stable performance under attention.	Social evaluation and responsibility pressure. Progressive difficulty and repetitive failure.	Emotion control, positive cognition, interpersonal assistance
	Basic tactical cooperation	Application: Assign students key tactical roles, whose execution quality is evaluated jointly by the team; suddenly require the next attack to be completed using specific tactics during practice matches. Purpose: To cultivate team adaptability to take responsibility and quickly execute instructions in dynamic collaboration.	Social evaluation and responsibility pressure. Goal obstacles and uncertainty.	Goal focus, interpersonal assistance
Gymnastics	Forward roll, shoulder stand	Application: Set precision challenges (drawing gradually narrowing movement channels), height challenges (adding thin mats to raise the movement plane), and pair trust exercises (one person practices, the other serves as the primary spotter). Purpose: Under the premise of safety, controllably trigger the worry about movement failure, guide students to shift their attention from “fear” to “technical essentials,” and learn peer trust and responsibility.	Risk perception and fear management. Goal obstacles and uncertainty. Social evaluation and responsibility pressure.	Emotion control, positive cognition, interpersonal assistance
Senior high school entrance examination items	One-minute rope skipping	Application: Compound fatigue interference (5 push-ups after 30 s of continuous jumping, then jump for the final 30 s), rhythm interference (skipping with irregular music), limit challenge (breaking personal best records). Purpose: To train the willpower to maintain internal rhythm and resist the urge to give up under the dual pressure of physical limits and external interference.	Physiological-cognitive dual interference. Progressive difficulty and repetitive failure.	Emotion control, goal focus

To ensure the scientificity and rationality of the experimental program, 8 experts in relevant fields were invited to evaluate the validity of the teaching experiment program, including 1 professor and 1 associate professor of physical education and training from a university, 1 chief physician and 1 associate chief physician from a hospital’s mental health center, 1 deputy director of the county education and sports bureau, 1 middle school party secretary (senior teacher, professor level), 1 middle school principal (senior teacher, associate professor level), and 1 middle school physical education teacher.

Main results of expert interviews: (1) All experts considered the overall experimental design reasonable, with 3 experts rating it “very reasonable,” 4 “reasonable,” and 1 “average”; (2) Regarding teaching content and scenario design, 4 experts rated it “very reasonable,” 3 “reasonable,” and 1 “average”; (3) The main modification suggestions from experts included: appropriately reducing the initial difficulty of some frustration scenarios, increasing specific guidance on emotion regulation skills, and strengthening individualized tutoring for underachieving students. The experimental program was revised and improved based on expert suggestions.

#### 3.2.2 Questionnaire survey method

The Resilience Scale for Chinese Adolescents (RSCA) developed by Hu and Gan (2008) was used as the assessment tool. The scale consists of 27 items divided into 5 dimensions, using a 5-point Likert scale. In this study, the overall Cronbach’s *α* coefficient of the scale was 0.764, and the Cronbach’s α coefficients of each dimension ranged from 0.734 to 0.768, indicating good reliability of the scale. Questionnaires were administered collectively to both groups before the intervention (week 1) and after the intervention (week 16). Paper questionnaires were distributed and collected on-site. The effective response rate for both the pre-test and post-test was 100%.

#### 3.2.3 Mathematical statistics method

All data were processed and analyzed using SPSS 29.0 statistical software. Measurement data were expressed as “mean ± standard deviation (M ± SD).” The statistical analysis process was as follows: Independent-samples t-tests and chi-square tests were used to test the homogeneity of baseline data between the two groups; Paired-samples t-tests were used to test intra-group differences in psychological resilience scores before and after the experiment for both groups, and Cohen’s *d* effect sizes were calculated (*d* < 0.2 = small effect, 0.2 ≤ *d* < 0.5 = medium effect, *d* ≥ 0.5 = large effect); Repeated-measures mixed ANOVA was used to test the Time × Group interaction effect; Analysis of covariance (ANCOVA) was used to test inter-group differences in post-test psychological resilience scores, with baseline scores as covariates, and partial η^2^ effect sizes were calculated (partial η^2^ < 0.01 = small effect, 0.01 ≤ partial η^2^ < 0.06 = medium effect, partial η^2^ ≥ 0.06 = large effect); The significance level was set at *α* = 0.05, where *p* < 0.05 indicated statistically significant differences, and *p* < 0.01 indicated highly statistically significant differences.

## 4 Results

### 4.1 Difference tests of psychological resilience between the two groups before and after the experiment

Paired-samples *t*-tests were conducted to compare the total psychological resilience scores and scores of each dimension between the two groups before and after the experiment. The results are presented in Table 3.

**Table 3:** Comparison of each dimension and total score of psychological resilience between the experimental group and the control group (M ± SD).

Dimension	Group	*N*	Pre-experiment	Post-experiment	*t*	*p*	Cohen *d*
Emotion control	Experimental group	57	18.25 ± 4.16	20.14 ± 3.16	−5.760	<0.001	0.76
	Control group	57	17.82 ± 4.26	18.79 ± 4.52	−4.978	<0.001	0.66
	*t*		0.534	1.849			
	*p*		0.594	0.067			
	Cohen *d*		0.10	0.35			
Goal focus	Experimental group	57	16.61 ± 4.18	17.35 ± 2.83	−3.094	0.003	0.41
	Control group	57	16.79 ± 4.73	16.95 ± 4.49	−2.421	0.019	0.32
	*t*		−0.21	0.575			
	*p*		0.834	0.567			
	Cohen *d*		0.04	0.30			
Interpersonal assistance	Experimental group	57	19.35 ± 4.67	20.02 ± 4.18	−5.399	<0.001	0.72
	Control group	57	18.68 ± 5.87	18.91 ± 5.48	−1.786	0.079	0.24
	*t*		0.671	1.211			
	*p*		0.503	0.228			
	Cohen *d*		0.13	0.23			
Positive cognition	Experimental group	57	14.60 ± 3.54	15.35 ± 2.28	−2.426	0.018	0.32
	Control group	57	13.84 ± 4.09	14.18 ± 3.91	−3.199	0.002	0.42
	*t*		1.053	1.961			
	*p*		0.294	0.052			
	Cohen *d*		0.20	0.37			
Family support	Experimental group	57	19.61 ± 4.55	19.58 ± 3.76	0.142	0.888	0.02
	Control group	57	18.18 ± 4.99	18.33 ± 4.93	−1.764	0.083	0.23
	*t*		1.607	1.517			
	*p*		0.111	0.132			
	Cohen *d*		0.30	0.28			
Total psychological resilience score	Experimental group	57	88.42 ± 13.34	92.44 ± 9.25	−5.052	<0.001	0.67
	Control group	57	85.32 ± 16.01	87.16 ± 15.46	−7.945	<0.001	1.05
	*t*		1.125	2.213			
	*p*		0.263	0.029			
	Cohen *d*		0.21	0.42			

*p* > 0.05 indicates no statistically significant difference; *p* < 0.05 indicates a statistically significant difference; *p* < 0.01 indicates a highly statistically significant difference.

Intra-group differences in the experimental group: After the intervention, the total psychological resilience score of students in the experimental group improved significantly compared with the pre-test (*t* = −5.052, *p* < 0.001, *d* = 0.67). In terms of dimensions, scores on Emotion Control (*t* = −5.760, *p* < 0.001, *d* = 0.76), Goal Focus (*t* = −3.094, *p* = 0.003, *d* = 0.41), Interpersonal Assistance (*t* = −5.399, *p* < 0.001, *d* = 0.72), and Positive Cognition (*t* = −2.426, *p* = 0.018, *d* = 0.32) all increased significantly, while no significant change was observed in the Family Support dimension (*p* > 0.05). Intra-group differences in the control group: After the intervention, the total psychological resilience score of students in the control group also improved significantly compared with the pre-test (*t* = −7.945, *p* < 0.001, *d* = 1.05). In terms of dimensions, scores on Emotion Control (*t* = −4.978, p < 0.001, *d* = 0.66), Goal Focus (*t* = −2.421, *p* = 0.019, *d* = 0.32), and Positive Cognition (*t* = −3.199, *p* = 0.002, *d* = 0.42) increased significantly, while no significant changes were found in Interpersonal Assistance and Family Support dimensions (*p* > 0.05).

Independent-samples t-tests were conducted to compare the post-test total psychological resilience scores and scores of each dimension between the experimental and control groups. The results are presented in Table 3. The results showed that after the intervention, the total psychological resilience score of the experimental group was significantly higher than that of the control group, and the difference was statistically significant (*t* = 2.213, *p* = 0.029, d = 0.42), confirming research hypothesis H3. In terms of dimensions, the differences between the two classes did not reach statistical significance (p > 0.05), but students in the experimental group had higher scores on all dimensions (Emotion Control, Goal Focus, Interpersonal Assistance, Positive Cognition, and Family Support) than those in the control group.

### 4.2 Repeated-measures mixed ANOVA of psychological resilience between the two groups before and after the experiment

A repeated-measures mixed ANOVA was conducted with the total psychological resilience score as the dependent variable, group as the between-subjects factor, and time as the within-subjects factor. The results are presented in Table 4. The results showed: The main effect of time was significant (*F* = 50.032, *p* < 0.001, partial η^2^ = 0.309), indicating that the total psychological resilience scores of both groups changed significantly before and after the experiment. The Time × Group interaction effect was significant (*F* = 6.896, *p* = 0.010, partial η^2^ = 0.058), indicating that there was a significant difference in the magnitude of improvement in total psychological resilience scores between the two groups, with the experimental group showing a significantly greater improvement than the control group. The main effect of group was not significant (*F* = 2.711, *p* = 0.102, partial η^2^ = 0.024), indicating no significant difference in the overall level of psychological resilience between the two groups, which was consistent with the previous baseline homogeneity test results.

**Table 4:** Repeated-measures mixed ANOVA of psychological resilience between the two groups before and after the experiment.

Source			Type III sum of squares	df	Mean square	*F*	Sig.	Partial eta squared
Within-subjects effects	Time	Sphericity assumed	489.281	1	489.281	50.032	<0.001	0.309
		Greenhouse–Geisser	489.281	1	489.281	50.032	<0.001	0.309
		Huynh-Feldt	489.281	1	489.281	50.032	<0.001	0.309
		Lower-bound	489.281	1	489.281	50.032	<0.001	0.309
	Time * Group	Sphericity Assumed	67.439	1	67.439	6.896	0.01	0.058
		Greenhouse–Geisser	67.439	1	67.439	6.896	0.01	0.058
		Huynh-Feldt	67.439	1	67.439	6.896	0.01	0.058
		Lower-bound	67.439	1	67.439	6.896	0.01	0.058
	Error (Time)	Sphericity assumed	1095.281	112	9.779			
		Greenhouse–Geisser	1095.281	112	9.779			
		Huynh-Feldt	1095.281	112	9.779			
		Lower-bound	1095.281	112	9.779			
Between-subjects effects	Intercept		1779033.333	1	1779033.333	4812.781	<0.001	0.977
	Group		1002.123	1	1002.123	2.711	0.102	0.024
	Error		41400.544	112	369.648			

### 4.3 Analysis of covariance (ANCOVA) of post-test psychological resilience between the two groups

To control for the influence of baseline scores on the experimental results, an analysis of covariance (ANCOVA) was conducted with the pre-test total psychological resilience score as the covariate and the post-test total psychological resilience score as the dependent variable. The results are presented in Table 5. The results showed that the overall model fit was good, with *R*^2^ = 0.922 and adjusted *R*^2^ = 0.921. The main effect of the covariate was significant, indicating that the pre-test score had a significant effect on the post-test score (*F* = 1256.362, *p* < 0.001). After controlling for the influence of the pre-test total psychological resilience score, the post-test total psychological resilience score of the experimental group was significantly higher than that of the control group (*F* = 15.551, *p* < 0.001), further confirming research hypothesis H3.

**Table 5:** Analysis of covariance (ANCOVA) of post-test psychological resilience between the two groups (tests of between-subjects effects; dependent variable: post-test psychological resilience).

Source	Type III sum of squares	df	Mean Square	*F*	Sig.
Corrected model	17491.220^a^	2	8745.61	658.082	<0.001
Intercept	996.311	1	996.311	74.97	<0.001
Group	206.661	1	206.661	15.551	<0.001
Pre-test psychological resilience	16696.474	1	16696.474	1256.362	<0.001
Error	1475.14	111	13.29		
Total	938,231	114			
Corrected total	18966.36	113			

^a^*R*^2^ = 0.922 (Adjusted *R*^2^ = 0.921).

### 4.4 Analysis of intervention effects among students of different genders

To further analyze the difference in the intervention effect of situational frustration education on junior high school students of different genders, t-tests were conducted on the total psychological resilience scores and scores of each dimension before and after the experiment for male and female students in the experimental group, respectively. The results are presented in Table 6.

**Table 6:** Comparison of each dimension and total score of psychological resilience between male and female students in the experimental group before and after the experiment (*M* ± SD).

Dimension	Sex	*N*	Pre-experiment	Post-experiment	*t*	*p*	Cohen *d*
Emotion control	Male	29	19.07 ± 3.63	20.69 ± 2.79	−4.167	<0.001	0.75
	Female	28	17.39 ± 4.54	19.57 ± 3.46	−4.056	<0.001	0.75
	*t*		1.541	1.346			
	*p*		0.129	0.184			
	Cohen *d*		0.40	0.35			
Goal focus	Male	29	17.00 ± 4.18	17.69 ± 3.13	−2.415	0.023	0.44
	Female	28	16.21 ± 4.22	17.00 ± 2.48	−2.017	0.054	0.37
	*t*		0.706	0.92			
	*p*		0.483	0.362			
	Cohen *d*		0.18	0.24			
Interpersonal assistance	Male	29	18.76 ± 4.63	19.52 ± 4.00	−3.863	<0.001	0.70
	Female	28	19.96 ± 4.71	20.54 ± 4.38	−3.827	<0.001	0.70
	*t*		−0.974	−0.918			
	*p*		0.334	0.363			
	Cohen *d*		0.26	0.24			
Positive cognition	Male	29	14.10 ± 3.77	15.07 ± 2.39	−1.765	0.088	0.32
	Female	28	15.11 ± 3.26	15.64 ± 2.16	−1.856	0.074	0.34
	*t*		−1.073	−0.949			
	*p*		0.288	0.347			
	Cohen *d*		0.28	0.25			
Family support	Male	29	19.83 ± 3.83	20.14 ± 3.58	−1.301	0.204	0.24
	Female	28	19.39 ± 5.27	19.00 ± 3.92	0.904	0.374	0.17
	*t*		0.357	1.145			
	*p*		0.722	0.257			
	Cohen *d*		0.09	0.30			
Total psychological resilience score	Male	29	88.76 ± 10.83	93.10 ± 8.11	−4.376	<0.001	0.79
	Female	28	88.07 ± 15.72	91.75 ± 10.41	−2.905	0.007	0.53
	*t*		0.193	0.549			
	*p*		0.848	0.586			
	Cohen *d*		0.05	0.14			

*p* > 0.05 indicates no statistically significant difference; *p* < 0.05 indicates a statistically significant difference; *p* < 0.01 indicates a highly statistically significant difference.

Cohen’s *d* systematically overestimates effect sizes when the sample size n is less than 30. Hedges’ g eliminates this bias through a correction factor, therefore Hedges’ g is reported for all effect size values in this study.

Paired-samples *t*-test results: Male students in the experimental group showed a highly significant improvement in total psychological resilience score after the experiment compared with the pre-test (*t* = −4.376, *p* < 0.001, Hedges’ *g* = 0.79). In terms of dimensions, the most significant improvements were observed in Emotion Control (*t* = −4.167, *p* < 0.001, Hedges’ *g* = 0.75) and Interpersonal Assistance (*t* = −3.863, *p* < 0.001, Hedges’ *g* = 0.70), both reaching large effect sizes. The Goal Focus dimension also showed a significant improvement (*t* = −2.415, *p* = 0.023, Hedges’ *g* = 0.44), reaching a medium effect size. No significant changes were found in Positive Cognition and Family Support dimensions (*p* > 0.05). Female students in the experimental group also showed a highly significant improvement in total psychological resilience score after the experiment compared with the pre-test (*t* = −2.905, *p* = 0.007, Hedges’ *g* = 0.53). In terms of dimensions, the most significant improvements were observed in Emotion Control (*t* = −4.056, *p* < 0.001, Hedges’ *g* = 0.75) and Interpersonal Assistance (*t* = −3.827, p < 0.001, Hedges’ *g* = 0.70), both reaching large effect sizes. Goal Focus (*t* = −2.017, *p* = 0.054) and Positive Cognition (*t* = −1.856, *p* = 0.074) showed increasing trends but did not reach statistical significance. No significant change was observed in the Family Support dimension (*p* > 0.05).

Independent-samples t-test results: There were no statistically significant differences in the total psychological resilience score and scores of each dimension between male and female students after the experiment (all p > 0.05). Among them, the effect size for the total psychological resilience score was Hedges’ *g* = 0.14, which was a negligible effect.

The results indicated that integrating situational frustration education into physical education had significant improvement effects on the psychological resilience of both male and female junior high school students, and there was no significant gender difference in the intervention effect, confirming research hypothesis H4.

## 5 Discussion

### 5.1 Enhancement effect of situational frustration education integrated into physical education on overall psychological resilience of junior high school students

The results of this study showed that after 16 weeks of teaching intervention, the overall psychological resilience levels of students in both the experimental and control groups improved significantly compared with the pre-test. However, the magnitude of improvement in the experimental group was significantly greater than that in the control group, and the total psychological resilience score of the experimental group was significantly higher than that of the control group after the experiment. This result fully verified hypotheses H1 and H3 of this study.

From a theoretical perspective, there are five core reasons why integrating situational frustration education into physical education can significantly improve junior high school students’ psychological resilience: First, the intervention program of this study achieved deep integration of frustration education and physical education, giving full play to the natural carrier advantage of physical education. Physical education takes physical activity as the core, and students inevitably encounter authentic frustration experiences during skill learning and competitive matches. Such embodied and authentic frustration experiences are incomparable to traditional didactic frustration education (Zhang et al., 2021). Unlike the random and fragmented frustration experiences in conventional physical education, the program of this study systematically embedded frustration education into the entire teaching process, making every frustration experience a goal-oriented, guided, and feedback-driven educational process.

Second, this study combined indigenous frustration education theory with international Self-Determination Theory to construct a more scientific theoretical framework. SDT points out that moderate and controllable need frustration can promote individual adaptive development (Trigueros and García-Mas, 2025). The frustration scenario design of this study strictly followed the “moderation” principle, controlling the degree of frustration within students’ Zone of Proximal Development (ZPD), avoiding both insufficient exercise effect due to overly low difficulty and excessive need frustration due to overly high difficulty.

Third, the systematic design of situational frustration education realized the upgrade of frustration education from “fragmented experiences” to “systematic cultivation.” The situational frustration education curriculum system constructed in this study divided frustration scenarios into five hierarchical levels: from basic skill mastery frustration, to advanced rule mutation frustration and pressure scenario frustration, and then to high-level team responsibility frustration and extreme challenge frustration. It gradually guided students to adapt to and cope with frustration, conforming to the psychological development laws of junior high school students (Wu, 1994).

Fourth, the intervention program realized a complete closed loop of “frustration experience––cognitive reconstruction––skill acquisition––behavior reinforcement,” conforming to the development laws of psychological resilience. The process theory of psychological resilience holds that the improvement of psychological resilience is a dynamic process in which individuals continuously activate protective factors and adjust cognition and behavior in adversity (Richardson, 2002). Each lesson in this study followed a complete teaching process, guiding students to complete the transformation from “experiencing frustration” to “managing frustration.”

Fifth, the intervention program integrated a variety of effective teaching methods, fully stimulating students’ subjective initiative and improving their self-efficacy. Bandura’s social learning theory holds that self-efficacy is the core driving force for the improvement of individual psychological resilience (Bandura, 1977). Through methods such as group cooperation, role modeling, and hierarchical task design, this study effectively improved students’ self-efficacy and formed a virtuous cycle of “challenge––success––improved self-efficacy––greater willingness to accept challenges.”

Notably, this study found that conventional physical education can also significantly improve the overall psychological resilience of junior high school students, which is consistent with the research conclusion of Huang et al. (2025). In conventional physical education, students also encounter certain frustrations and challenges during skill learning and physical training, and the process of overcoming difficulties through repeated practice itself has a positive impact on students’ willpower. However, compared with conventional physical education, physical education integrated with situational frustration education has a more significant improvement effect on psychological resilience. The core reason is that the core goal of conventional physical education is skill teaching and physical fitness improvement, with insufficient attention to students’ psychological development, and frustration experiences are random and unsystematic. In contrast, situational frustration education takes improving psychological resilience as one of the core teaching goals, systematically designs frustration scenarios, and carries out targeted frustration resistance skill training, realizing the deep integration of “physical education” and “mental education.”

### 5.2 Differential effects of situational frustration education integrated into physical education on various dimensions of psychological resilience

The results of this study showed that after the intervention, the scores of four dimensions in the experimental group—Emotion Control, Goal Focus, Interpersonal Assistance, and Positive Cognition—all improved significantly compared with the pre-test, while no significant change was observed in the Family Support dimension. This result verified hypothesis H2 of this study.

The Emotion Control dimension was one of the dimensions with the largest improvement in the experimental group, with a highly significant increase in scores after the experiment. This result is highly consistent with the research expectation and also reflects the core effect of situational frustration education. Junior high school students are in early adolescence, and the development of the hypothalamic–pituitary–adrenal (HPA) axis is not yet mature, resulting in emotions characterized by high volatility and impulsivity (Hu and Gan, 2008). The situational frustration education program of this study specifically integrated systematic psychological skill training, setting up an emotion regulation session at the end of each class to teach students practical emotion regulation skills such as deep breathing relaxation and positive self-talk. During the 16-week teaching period, students repeatedly experienced the process of “negative emotion arousal––application of regulation strategies––emotional calming,” gradually internalizing the acquired emotion regulation skills into stable behavioral habits.

The scores of the Goal Focus dimension also improved significantly after the experiment. In the situational frustration education program of this study, all frustration tasks were designed around clear skill goals, guiding students to always focus on core goals when facing frustrations and difficulties, learn to break down large goals into small ones, and gradually overcome difficulties and achieve goals through planning and step-by-step implementation (Xin, 1999). Meanwhile, teachers guide students to shift their attention from the negative emotion of “I failed” to the problem-solving of “how to adjust movements and complete goals” after task failure, preventing students from giving up goals due to frustration (Yu, 2007).

The Interpersonal Assistance dimension was another dimension with a relatively large improvement in the experimental group, while no significant change was observed in the control group before and after the experiment. This is one of the most prominent advantages of situational frustration education compared with conventional teaching. This result is highly consistent with the survey results of Shao et al. (2022), who found that when junior high school students encounter frustration in physical education, 61.4% of them choose to ask classmates for help, and peers are the primary source of support for junior high school students to cope with frustration. The situational frustration education program of this study extensively adopted forms such as group cooperative learning and team competitive matches, and many frustration tasks required the joint collaboration of group members to complete. In such scenarios, students learned how to communicate and collaborate with peers, how to provide support and encouragement to peers when they encounter frustration, and how to ask peers for help when they themselves encounter difficulties, gradually improving their interpersonal communication skills and social support utilization ability (Xia et al., 2024).

The scores of the Positive Cognition dimension also improved significantly after the experiment. Positive cognition refers to the cognitive mode in which individuals can dialectically view frustration and discover growth opportunities from adversity. It is the cognitive core of psychological resilience and the essential goal of frustration education (Yu, 2007). In the teaching program of this study, after each frustration task, teachers guide students to reflect not only on motor skill problems but also on “What did you learn from this failure?” and “How has this frustration helped your growth?.” Through repeated guidance, students are helped to reconstruct their cognition of frustration, transforming from the negative cognition that “frustration is a bad thing” to the positive cognition that “frustration is an opportunity for growth.”

No significant changes were observed in the Family Support dimension in both the experimental and control groups before and after the experiment, which is consistent with the research expectation. The core reasons for this result are mainly twofold: First, the teaching intervention of this study was carried out entirely in school physical education classes, and the intervention content mainly focused on students’ frustration coping in school scenarios, without involving interventions related to family environment, parent–child communication, and family support systems, making it difficult to have a significant impact on students’ Family Support dimension. Second, the Family Support dimension measures students’ perception of family support, and the scores of this dimension are mainly affected by long-term family factors such as family parenting style, parent–child relationship, and family atmosphere. Short-term school physical education interventions are difficult to produce significant changes in this dimension (Zhang, 2019). This result also reminds us that the cultivation of adolescent psychological resilience cannot rely solely on school education; a home-school-community collaborative education system must be constructed to achieve all-round and all-dimensional improvement.

### 5.3 Difference analysis of intervention effects among students of different genders

The results of this study showed that integrating situational frustration education into physical education had significant improvement effects on the psychological resilience of both male and female junior high school students, and there was no significant gender difference in the improvement effect. This result differs from some existing studies. Shao et al. (2022) found that junior high school girls have significantly higher frustration experience in physical education than boys, and girls have relatively weaker frustration tolerance.

The core reason for the results of this study lies in the fact that the situational frustration education program fully followed the principle of individualized teaching and set differentiated frustration scenario difficulties and task types according to the physical and psychological characteristics of male and female students. For example, in physical fitness items, higher intensity challenges were set for boys based on their physical fitness advantages, while challenges focusing more on rhythm control and movement accuracy were set for girls based on their physical characteristics. This avoided both excessive frustration in girls due to overly high difficulty and insufficient exercise effect in boys due to overly low difficulty. Meanwhile, in terms of event selection, the program balanced competitive and confrontational sports preferred by boys and rhythmic and collaborative sports preferred by girls, allowing both male and female students to gain successful experiences in their areas of expertise while accepting moderate frustration challenges in areas they are not good at.

A study by Kegelaers and Wylleman (2019) also pointed out that personalized and differentiated intervention programs are the key to improving the effect of frustration resistance education and can effectively avoid intervention effect deviations caused by gender and individual differences. The results of this study confirm that as long as the principle of individualized teaching is followed and frustration scenarios are designed in line with students’ physical and psychological characteristics, integrating situational frustration education into physical education can have significant positive effects on the psychological resilience of both male and female junior high school students, with good universality.

### 5.4 Analysis of the reasons for significant improvement in the control group

This study found that students in the control group also showed significant improvements in total psychological resilience scores and scores of Emotion Control, Goal Focus, and Positive Cognition dimensions without receiving situational frustration education intervention. This result requires in-depth analysis. Possible reasons include the following three aspects: First, the maturation effect. The experimental period of this study was 16 weeks, during which junior high school students are in a stage of rapid physical and psychological development, and their emotion regulation ability, goal focus ability, etc., will naturally improve with age. Second, the Hawthorne effect. Since students knew they were participating in a study, they would pay more attention to their behavioral performance and be more engaged in physical education classes, thereby having a positive impact on psychological resilience (Sedgwick and Greenwood, 2015). Third, the positive effect of conventional physical education. As mentioned earlier, conventional physical education itself has a certain mental health promotion effect. The natural frustrations encountered by students during skill learning and physical training, as well as teachers’ regular guidance, also have a positive impact on students’ psychological resilience.

It should be noted that although the control group also showed significant improvement, the magnitude of improvement in the experimental group was significantly greater than that in the control group, which fully demonstrates the unique value of situational frustration education intervention.

### 5.5 Theoretical contributions and practical implications of this study

#### 5.5.1 Theoretical contributions

The theoretical contributions of this study are mainly reflected in three aspects: First, this study combined indigenous frustration education theory with international Self-Determination Theory to construct a more explanatory theoretical framework. For the first time, this study introduced SDT into situational frustration education research and proposed that “moderate need frustration” is the core mechanism for promoting the development of psychological resilience, enriching and expanding the theoretical application system of frustration education. Second, this study deepened the research on the mental health promotion mechanism of physical education. Through rigorous teaching experiments, this study revealed the internal path through which situational frustration education improves overall psychological resilience by enhancing students’ abilities in emotion control, goal focus, interpersonal assistance, and positive cognition, clarifying the mechanism of “physical education––frustration experience––frustration resistance skill acquisition––psychological resilience improvement.” Third, this study supplemented relevant research on psychological resilience intervention for junior high school students. Taking junior high school students as the research object, this study verified the intervention effect of situational frustration education on junior high school students’ psychological resilience through a 16-week teaching experiment and analyzed its differential effects on various dimensions, providing a new perspective and empirical evidence for the cultivation of junior high school students’ psychological resilience.

#### 5.5.2 Practical implications

The results of this study have important practical implications for junior high school physical education and adolescent mental health education: First, junior high school physical education should break through the traditional teaching model of “valuing skills over psychology” and deeply integrate mental health education into the entire process of physical education. Physical education teachers should fully recognize the “mental education” value of physical education. In daily teaching, they should not only pay attention to students’ skill mastery and physical fitness improvement but also focus on their psychological development, and incorporate frustration education, emotion regulation, and willpower cultivation into teaching goals. Second, when carrying out frustration education in physical education, situational and systematic design should be adopted to avoid fragmented and deliberate frustration settings. Physical education teachers should design hierarchical, authentic, and interactive frustration scenarios around teaching content in accordance with students’ physical and psychological development laws, and embed frustration education into the entire process of skill teaching. Third, the implementation of frustration education should follow the principles of moderation and individualized teaching to realize the organic combination of frustration education and success education. When designing frustration scenarios, physical education teachers should fully consider students’ individual differences to ensure that frustration challenges are within students’ ZPD. Meanwhile, when students encounter frustration, they should provide sufficient support, encouragement, and guidance to help students finally overcome difficulties and gain successful experiences. Fourth, a home-school-community collaborative adolescent frustration education system should be constructed to realize all-round psychological resilience cultivation. The results of this study show that school physical education interventions have no significant effect on students’ Family Support dimension, which reminds us that the cultivation of adolescent psychological resilience cannot rely solely on school education. Schools should strengthen collaborative cooperation with families and communities, guide parents to establish correct frustration education concepts, and carry out rich practical activities in conjunction with communities to build a comprehensive home-school-community collaborative frustration education system.

### 5.6 Research limitations and future prospects

Although this study verified the improvement effect of situational frustration education integrated into physical education on junior high school students’ psychological resilience through rigorous teaching experiments, there are still some limitations: First, the representativeness of the research sample is limited. This study only selected Grade 8 students from one middle school in Luzhou City, Sichuan Province as the research object. The geographical and grade ranges of the sample are relatively narrow, which limits the generalizability of the research results to a certain extent. Second, there are limitations of the cluster randomization design. This study adopted the cluster sampling method and randomly assigned groups by class rather than by individual, which may lead to clustering effects and affect the internal validity of the study. Future research can adopt a multicenter cluster randomized controlled trial to reduce the impact of clustering effects. Third, there is a potential influence of experimenter bias. Although this study adopted standardized teaching procedures and classroom monitoring, it is still impossible to blind the teaching instructor, which may lead to certain experimenter bias (Edlund et al., 2021). Future research can adopt the method of multiple teachers teaching separately to reduce the impact of experimenter bias. Fourth, there are limitations of the single measurement method. This study only used self-report questionnaires to measure psychological resilience, which may have common method variance bias and social desirability bias. Future research can combine multiple measurement methods such as teacher evaluation and behavioral observation to improve measurement accuracy. Fifth, the experimental intervention period and follow-up are insufficient. The experimental intervention period of this study was 16 weeks, which only tested the immediate effect of the intervention. No long-term follow-up was conducted on students, so the long-term sustainability of the intervention effect cannot be determined.

In view of the limitations of this study, future research can be deepened and expanded from the following five aspects: First, expand the research sample range and carry out multicenter and large-sample empirical studies to further verify the intervention effect of situational frustration education integrated into physical education and improve the generalizability of the research results. Second, adopt more rigorous research designs, such as multicenter cluster randomized controlled trials, to reduce the impact of clustering effects and experimenter bias. Third, adopt multiple measurement methods, combining self-report, teacher evaluation, and behavioral observation, to comprehensively evaluate the intervention effect. Fourth, extend the experimental period and carry out long-term follow-up studies to test the long-term sustainability and stability of the intervention effect. Fifth, construct a home-school-community collaborative situational frustration education system, incorporate family and social level interventions into the research design, and test their improvement effects on all dimensions of adolescent psychological resilience.

## 6 Conclusions and recommendations

### 6.1 Conclusion

By constructing a curriculum system integrating situational frustration education into junior high school physical education and conducting a 16-week parallel controlled teaching experiment with 114 junior high school students as the research object, this study systematically examined its intervention effect on junior high school students’ psychological resilience and drew the following conclusions:

Integrating situational frustration education into physical education can significantly improve the overall psychological resilience of junior high school students, and its improvement effect is significantly better than that of conventional junior high school physical education.

Integrating situational frustration education into physical education has differential improvement effects on different dimensions of junior high school students’ psychological resilience. It can significantly enhance students’ abilities in emotion control, goal focus, interpersonal assistance, and positive cognition, but has no significant improvement effect on the Family Support dimension.

Conventional junior high school physical education can also significantly improve the overall psychological resilience of junior high school students, as well as their abilities in emotion control, goal focus, and positive cognition, but has no significant improvement effect on interpersonal assistance and family support dimensions.

Integrating situational frustration education into physical education has significant improvement effects on the psychological resilience of both male and female junior high school students, with no significant gender difference in the improvement effect, showing good universality.

### 6.2 Recommendations

Based on the conclusions of this study and combined with the actual situation of junior high school physical education, the following recommendations are proposed:

Educational administrative departments should strengthen top-level design, incorporate frustration education and mental health education into the core teaching goals of junior high school physical education and health courses, issue relevant teaching guidance documents, and organize special training for physical education teachers to improve their mental health education ability and frustration education implementation ability.

Junior high school physical education teachers should transform their teaching concepts, fully recognize the “mental education” value of physical education, break the traditional teaching model of “valuing skills over psychology,” systematically integrate situational frustration education into daily physical education, design hierarchical frustration scenarios around teaching content, and construct a teaching closed loop of “skill learning––frustration experience––cognitive reconstruction––skill acquisition––behavior reinforcement.”

Schools should construct a collaborative education mechanism between physical education and mental health education, strengthen cooperation between physical education teachers and psychological teachers, and jointly design frustration education curriculum programs. Meanwhile, through parent schools and home-school communication meetings, popularize the concepts and methods of frustration education to parents and guide them to cooperate with schools in carrying out frustration education.

Junior high school students themselves should actively participate in sports activities, proactively face frustrations and challenges in physical education learning, temper their willpower in the process of overcoming difficulties, actively learn relevant skills of emotion regulation and problem-solving, and learn to seek help from peers, teachers, and parents.

## Data Availability

The original contributions presented in the study are included in the article/supplementary material, further inquiries can be directed to the corresponding author.
